# Curative Radiotherapy for Locally Advanced Scalp Squamous Cell Carcinoma

**DOI:** 10.7759/cureus.18514

**Published:** 2021-10-05

**Authors:** Daniel Xing, Supan Hettige, Lessandra Yan Shan Chee, Rohan Nair, Rajendra Hegde

**Affiliations:** 1 Radiation Oncology, Olivia Newton-John Cancer Wellness and Research Centre, Austin Health, Heidelberg, AUS; 2 Gippsland Radiation Oncology, Latrobe Regional Hospital, Traralgon, AUS; 3 Radiation Oncology, William Buckland Radiotherapy Centre, The Alfred Health, Melbourne, AUS

**Keywords:** external beam radiation, head and neck cancer, volumetric-modulated arc therapy, radiotherapy, cutaneous squamous cell carcinoma

## Abstract

A 73-year-old man presented to his primary physician with an ulcerative growth on his scalp vertex. Biopsy of the lesion confirmed the growth to be a moderately differentiated squamous cell carcinoma, but the patient declined medical intervention. The lesion increased in size over six months, measuring 12 cm in diameter and 3 cm thickness with erosion of the skull of the vertex. CT and MRI scans showed a large fungating mass with erosion of the skull of vertex without intra-cranial extension, meningeal enhancement, or distant metastatic disease. The patient declined surgical intervention. The patient received radiotherapy using volumetric-modulated arc therapy (VMAT) to a total dose of 60 Gy over six weeks. No evidence of clinical invasive disease apart from a 15 cm * 12 cm skin defect detected three months after completion of radiotherapy. At three years of follow-up, the patient is clinically disease-free. This case report provides evidence that high-dose radiotherapy is a potential effective definitive treatment for locally advanced (T4) squamous cell carcinoma for patients who are unwilling to undergo surgery.

## Introduction

The incidence of cutaneous squamous cell carcinoma (cSCC) is high in Australia (271 per 100,000 person-years) [1]. Patients with T4 (i.e., tumor with cortical bone or marrow invasion) locally advanced cSCC usually need a major surgical procedure with associated morbidity, and adjuvant radiotherapy is usually required to provide the best possible disease outcome, although the curative rate is not high with multi-modality treatments for these patients [2]. Radiotherapy usually serves as an effective palliative treatment for patients with T4 locally advanced cSCC especially for elderly patients and patients with poor performance status. Here, we report a clinical case of a patient with a scalp T4 cSCC who was successfully treated with curative intent radiotherapy alone.

## Case presentation

**Figure 1:**
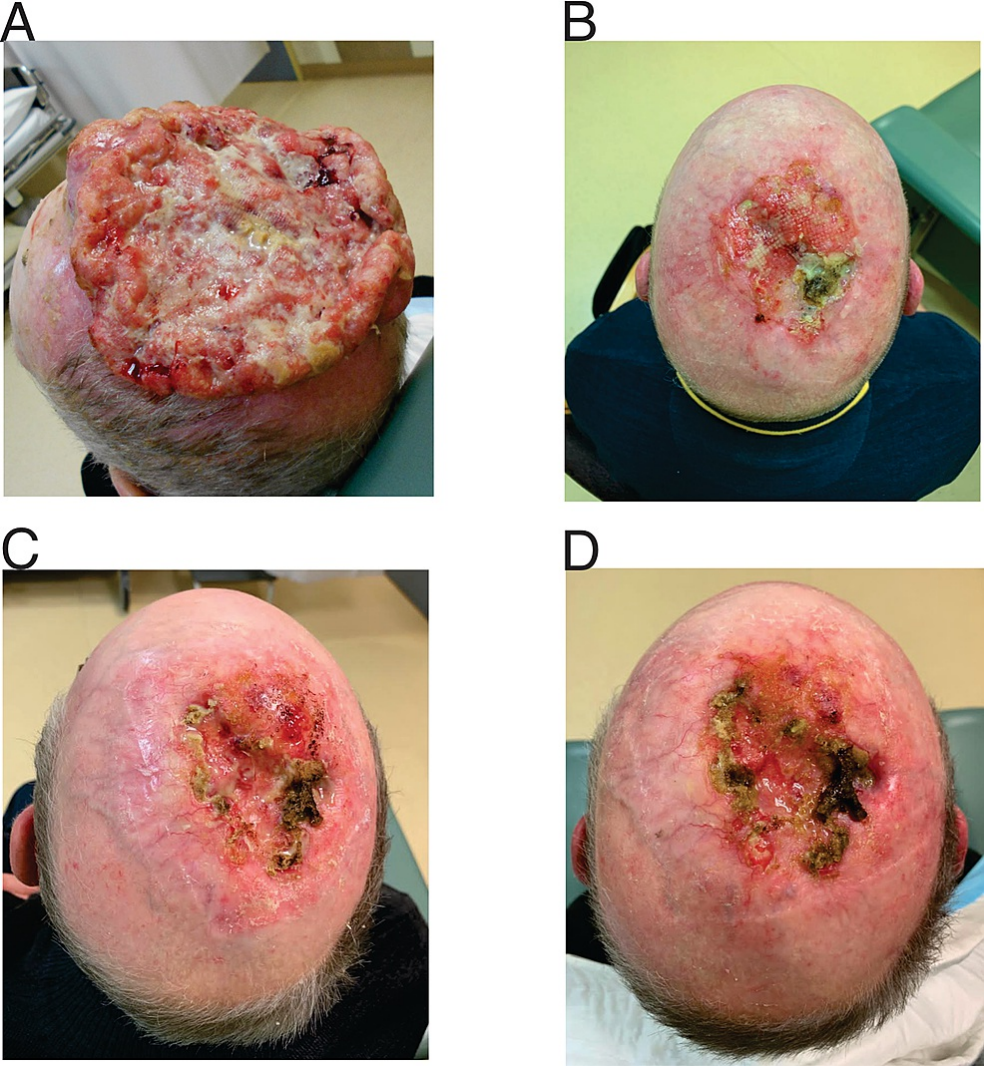
Clinical images of ulcerative growth on the scalp vertex with extensive central necrosis. (A) Presentation, (B) 12 weeks post-radiotherapy, (C) 28 months post-radiotherapy, and (D) 36 months post-radiotherapy

A 73-year-old man presented to his primary physician with a 4-cm ulcerative growth on his scalp vertex with extensive central necrosis in May 2018. Biopsy of the lesion confirmed moderately differentiated squamous cell carcinoma with no evidence of vascular or perineural invasion. It was resectable wide-local excision and flap reconstruction. The patient declined surgical intervention. The lesion increased in size over six months, measuring 12 cm in diameter and 3 cm in thickness (Figure 1, panel A).

**Figure 2:**
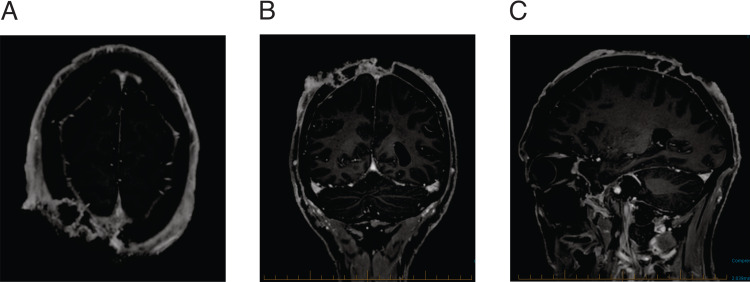
T1 MR image at presentation. (A) Axial image, (B) coronal image, and (C) sagittal image

The patient was referred to our center for consideration of radiotherapy since he was determined not to proceed with surgical resection. CT scan showed a 12-cm fungating mass at scalp vertex with erosion and destruction of the calvarial vault. There was no evidence of regional or distant metastatic disease on CT scan of neck, chest, and abdomen. The MRI scan again demonstrated a large fungating scalp mass with the erosion of the skull in the region of the vertex. The inner table appeared intact and no intracranial extension or meningeal enhancement was seen (Figure 2) (AJCC 8 stage IV; T4 N0 M0).

The patient received radiotherapy using volumetric-modulated arc therapy to a total dose of 60 Gy over six weeks. The patient tolerated the radiotherapy well, and the tumor reduced over 50% in size after three weeks of radiotherapy (i.e., 30 Gy in 15 fractions). We re-planned his radiotherapy at the fourth week of treatment due to the significant change in the target volume. The patient develops confluent moist desquamation, i.e., grade 3 radiation dermatitis as per Common Terminology Criteria for Adverse Events (CGCAE) Version 5.0. There was no treatment interruption due to the acute toxicity. Acute dermatitis resolved completely after six weeks of careful wound care. No evidence of invasive disease apart from a 15 cm * 12 cm skin defect was detected three months after completion of radiotherapy (Figure 1, panel B). The patient remains clinically disease-free at 28-month (Figure 1, panel C) and 36-month follow-up (Figure 1, panel D).

## Discussion

Radiotherapy plays an important role in managing cutaneous squamous cell carcinoma (cSCC) in both adjuvant and curative settings [3]. Although surgery is the standard of care for the majority of cSCC, definitive radiotherapy can be considered in patients with cSCC in anatomic locations where surgery can compromise function (e.g., ears, nose, lips, or eyelids) [2]. There is evidence that radiotherapy can provide high local control rates for cSCC as high as 90%, although most of the patients presented with T1-2 disease in those studies [4]. For T4 advanced cSCC, it is generally accepted that surgery followed by adjuvant radiotherapy offers better local control since radiotherapy alone can only achieve local control around 40-50% at four to five years in retrospective studies [5-7]. Hence, radiotherapy is usually regarded as a palliative intent treatment for surgically inoperable patients. On the other hand, curative surgery for T4 cSCC can be associated with high morbidity with poor cosmetic and function outcomes. Furthermore, adjuvant radiotherapy, which would have been indicated in this case, maybe poorly tolerated. Hence, careful patient selection is important in T4 cSCC. In a similar case of locally advanced cSCC reported by Papadopoulos et al., a combined craniotomy and very complex flap repair were required to achieve complete resection, but the patient developed local recurrence shortly without adjuvant radiotherapy [8].

**Figure 3:**
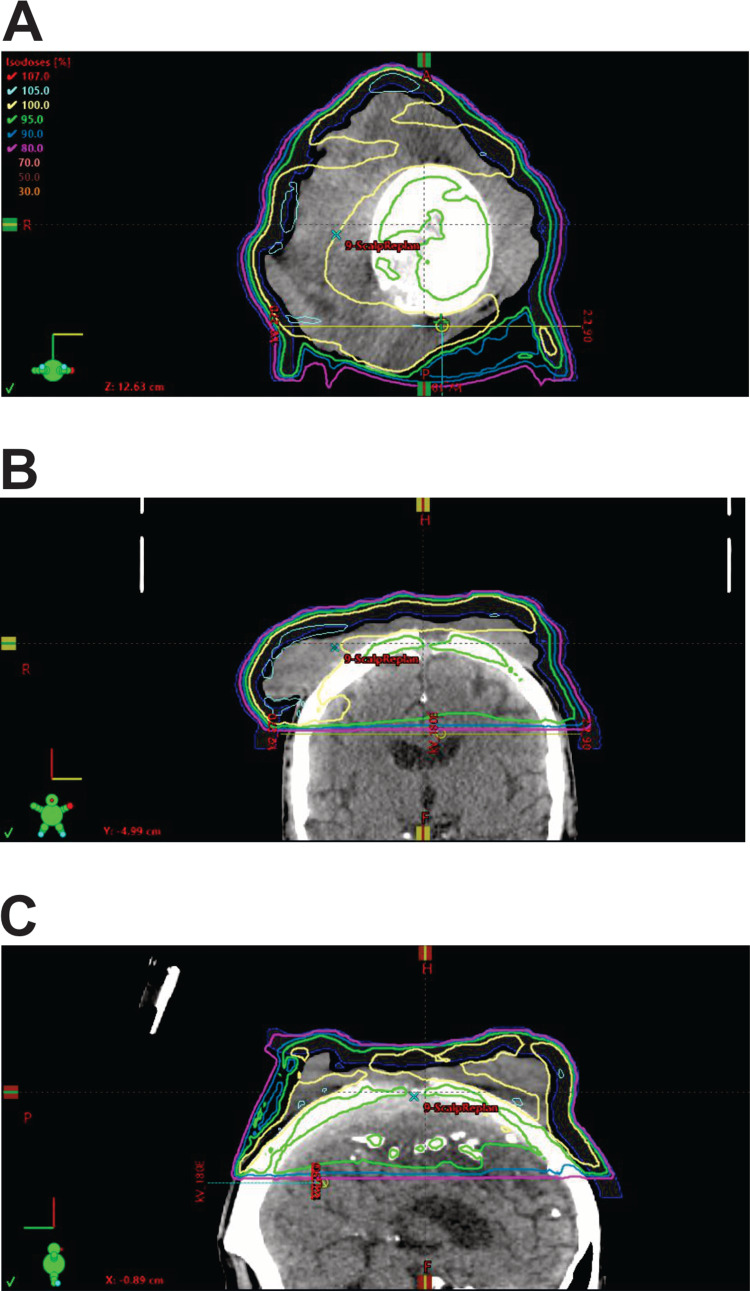
Radiotherapy plan for the patient. (A) Axial view, (B) coronal view, and (C) sagittal view (blue: 90% isodose line; green: 95% isodose line; yellow: 100% isodose line)

Various radiotherapy modalities can be used to treat cSCC including kilovoltage photons (i.e., superficial radiotherapy), megavoltage (MV) electrons, MV photons, and brachytherapy as well. With the advancement in radiotherapy techniques, volumetric-modulated arc therapy (VMAT) with MV photons radiotherapy has several advantages over the other external beam radiotherapy to treat large volume locally advanced cSCC. The radiotherapy dosimetry was highly conformal to ensure that the gross disease is well covered by the prescribed dose as shown in Figure 3.

It also allowed us to meet the relative dose constraints to the organs at risk, particularly in this case, the brain. We accepted the maximal dose of 49 Gy and the mean dose of 13 Gy in this case. We used a daily cone beam for treatment verification to ensure the setup accuracy. The elective nodal treatment for cSCC is based on estimated nodal relapse risk, and VMAT can deliver simultaneous nodal irradiation if it is indicated [2]. In our case, the elective nodal irradiation may be too extensive, and it can be potentially salvaged if the patient develops neck nodal metastasis. It has been reported that intensity-modulated radiotherapy (IMRT) is associated with 60-80% local control rate at 12 months in advanced cSCC and acceptable grade 3 toxicity tolerance in a retrospective study [9]. Brachytherapy would have been able to provide even better conformity whilst sparing brain dose but has logistic challenges. It is not widely available in Australia.

Patient characteristics are another important factor that is associated with outcome. Treatment with radiation alone is associated with poor disease-free survival in patients with locally advanced disease, older age, positive node, and immune dysfunction [7]. Patients with immunosuppression had worse outcomes with cSCC in general [10]. Our patient is relatively young with no comorbidities. He had excellent performance status prior to the presentation, but he declined any surgical intervention. It is still unknown how to select radioresponsive disease, and who would do well with radiotherapy alone in cSCC.

Cutaneous SCC has a high mutation burden [11] and the disease risk is strongly associated with immunosuppression [10]. Recent studies have shown that cSCC is responsive to immune therapy [12]. In phase 2 single-arm study, Migden et al. reported that patients had a 44% objective response rate (34/78) to cemiplimab, a programmed death 1 (PD-1) receptor blockade, with a median follow-up of 9.3 months [13]. However, only 10 patients (13%) had a complete response. Cemiplimab is also associated with 44% grade 3-4 adverse events, and 23 (29%) of 78 patients had serious treatment-related adverse events. Furthermore, one of the risk factors for cSCC is immunosuppression, which is also a contra-indication to immune therapy. Hence, we would argue that radiotherapy can be an effective treatment option for patients with localized T4 cSCC if feasible. Immune therapy can be reserved when the disease recurs or progresses.

## Conclusions

Cutaneous squamous cell carcinoma can be a radioresponsive disease. This case report provides evidence that high-dose radiotherapy can be an effective treatment option for T4 advanced cSCC. Advanced radiotherapy techniques (e.g., VMAT) enables delivery of high-dose radiotherapy to a large target volume with acceptable low-dose spread to normal tissues. It can be served as a potential option for patients who are not surgical candidates or decline surgical intervention. Further studies are required to evaluate the long-term outcome of definitive radiotherapy alone compared with radical surgical resection for locally advanced cSCC.
